# Medium-term health-related quality of life in patients with pulmonary arterial hypertension treated with goal-oriented sequential combination therapy based on exercise capacity

**DOI:** 10.1186/s12955-019-1178-x

**Published:** 2019-06-14

**Authors:** Akihiro Hirashiki, Shiro Adachi, Naoki Okumura, Yoshihisa Nakano, Shigetake Shimokata, Atsuya Shimizu, Hidenori Arai, Kenji Toba, Toyoaki Murohara, Takahisa Kondo

**Affiliations:** 1 Department of Cardiology, Nagoya University Graduate School of Medicine, Nagoya, 466-8560 Japan; 2 Department of Cardiology, National Center for Geriatrics and Gerontology, 7-430, Morioka-cho, Obu, Aichi, Morioka, 474-8511 Japan; 3 Department of Advanced Medicine in Cardiopulmonary Disease, Nagoya University Graduate School of Medicine, Nagoya, 466-8560 Japan

## Abstract

**Background:**

Pulmonary arterial hypertension (PAH) remains a life-threatening condition, despite modern therapies. We prospectively investigated the therapeutic health-related quality of life (HRQOL) effects of goal-oriented sequential combination therapy based on exercise capacity in patients newly diagnosed with PAH.

**Methods:**

To examine the changes in HRQOL in PAH patients, we treated 30 patients newly diagnosed with PAH with goal-oriented sequential combination therapy based on exercise capacity. We monitored exercise capacity by cardiopulmonary exercise testing and observed the benefit of using a peak VO_2_ cut-off of 15 mL/kg/min to guide combination therapy. First-line treatment was an endothelin receptor antagonist (ERA); second-line treatment was the addition of a phosphodiesterase-5 inhibitor (PDE-5I). At baseline and at 3, 6, and 12 months, HRQOL was evaluated by using the eight-item Medical Outcomes Survey Short Form Health Survey.

**Results:**

At 12 months, 100% of PAH patients were receiving an ERA, and 82% an ERA + PDE-5I. The mean physical component summary (PCS) score was 33.5 at baseline, 41.2 at 3 months, 40.8 at 6 months, and 42.0 at 12 months, and the mean mental component summary (MCS) scores were 45.6, 47.0, 50.0, and 50.1, respectively. PCS score was significantly greater at 3 months than at baseline (*P* = 0.035). MCS score was comparable at 3 months and at baseline, but was significantly greater at 6 and 12 months than at baseline (*P* = 0.033, *P* = 0.028, respectively). Thus, PCS score improved soon after initiation of therapy, and MCS score improved later.

**Conclusions:**

Together, these results suggest that goal-oriented sequential combination therapy based on exercise capacity improves HRQOL in patients with PAH.

## Introduction

Pulmonary arterial hypertension (PAH) is a life-threatening condition that is associated with poor prognosis [1], despite the availability of many therapeutic options. For the treatment of PAH, 3 different molecular pathways can be targeted with 5 families of drugs: prostanoids, prostanoid receptor agonists (i.e., selexipag), endothelin receptor antagonists (ERAs), phosphodiesterase-5 inhibitors (PDE-5Is), and soluble guanylate cyclase stimulators [2]. Current treatment algorithms for PAH recommend early use of either an ERA or a PDE-5I as first-line treatment in patients with World Health Organization (WHO) functional class II or III PAH [3]. Compared with monotherapy, combination therapy improves exercise capacity and reduces the risk of clinical worsening in PAH patients [3–6]. However, the optimum combination therapy remains controversial. There is increasing recognition of the value of cardiopulmonary exercise testing (CPX) for guiding treatment in patients with pulmonary hypertension [5, 7–10]. The 2015 European Society of Cardiology and European Respiratory Society (ESC/ERS) guidelines for the diagnosis and treatment of pulmonary hypertension associate a cut-off value of peak VO_2_ ≥ 15 mL/min/kg with a better prognosis [3].

There is also growing interest in the analysis of health-related quality of life (HRQOL) in patients with PAH [11, 12]. PAH imposes a considerable strain on patients and families in terms of prognosis and treatment-related difficulties. The 36-item Medical Outcomes Study Short Form Health Surveys (SF-36) are general, non-disease-specific instruments for assessing QOL and are considered useful for evaluating treatment efficacy in patients with pulmonary hypertension [13–15]. SF-36 correlates well with 6-min walking distance (6MWD) and WHO functional classification, but not with hemodynamic parameters [16]. Since the reliability and validity of the Japanese version of the SF-8 was confirmed in the general Japanese population [17], it has been used in several Japanese studies as a measure of HRQOL outcomes [18–20]. Using the Japanese version of the SF-8 allows us to compare HRQOL between patients with different diseases and to compare diseases with studies from other countries [21, 22].

It has been suggested that HRQOL is associated with prognosis in patients with PAH [23]. However, little is known about the changes in QOL over time in newly diagnosed PAH patients undergoing goal-oriented sequential combination therapy. Here, we prospectively investigated the therapeutic HRQOL effects of goal-oriented sequential combination therapy based on exercise capacity in patients newly diagnosed with PAH.

## Methods

### Study design and population

This prospective study was conducted at Nagoya University Hospital, Japan, from October 2012 through March 2015. Eligible patients were 16 to 80 years old at study entry and had newly diagnosed PAH of WHO functional classes II to IV according to the ESC/ERC guideline criteria [3]. Patients who had WHO functional class IV disease with hemodynamic instability were immediately treated with intravenous epoprostenol, if needed.

Patients with any of the following conditions at enrollment were excluded: 1) pulmonary hypertension corresponding to group 2, 3, 4, or 5 in the classification or PAH with congenital heart disease; 2) pregnancy; 3) serum creatinine > 2.0 mg/dL; 4) history of serious chronic obstructive pulmonary disease or restrictive lung disease; 5) inability to walk without personal assistance; 6) currently receiving PAH-targeted therapy such as an ERA, a PDE-5I, or intravenous epoprostenol; 7) any other condition that, by judgment of the physicians in charge, made enrollment inappropriate because of concerns for patient safety.

### Informed consent

The study protocol was approved by the Ethics Review Board of Nagoya University School of Medicine (approval no. 1157). After a physician-in-charge explained the study objectives, study protocol, possible adverse effects of PAH-targeted drugs, measures for privacy protection, and procedures for study withdrawal, all participants provided their written informed consent.

### Study procedures and medical protocol

**Fig. 1:**
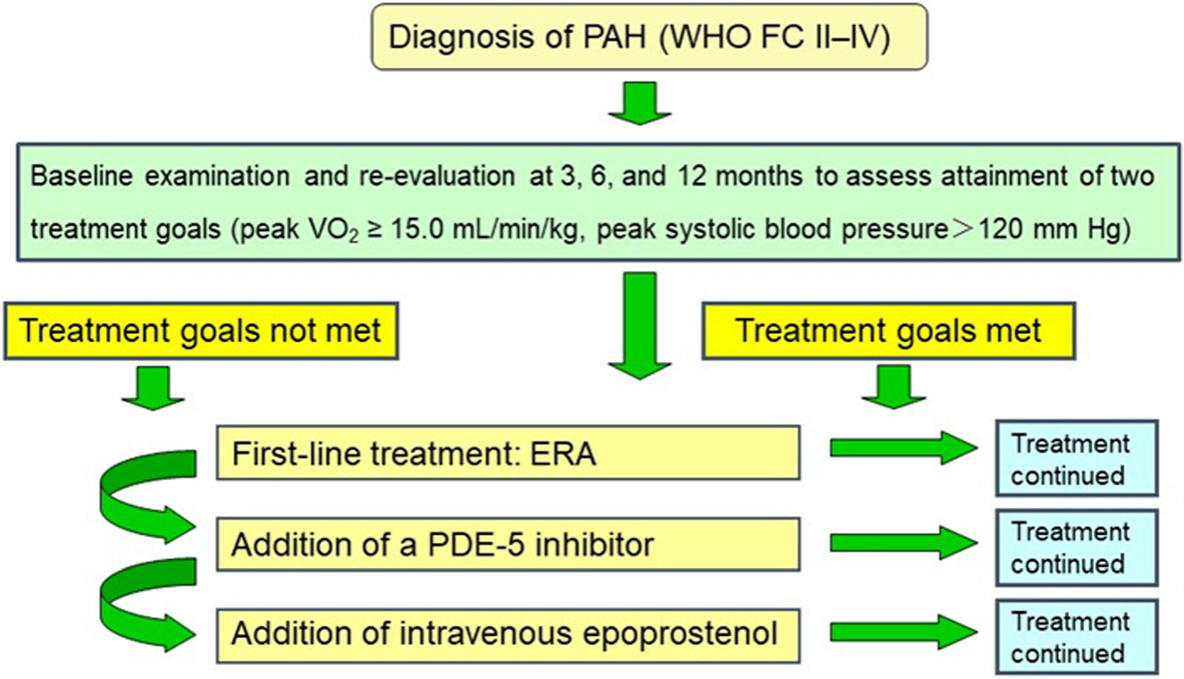
Study protocol. Goal-based therapeutic algorithm used in the present study for patients with newly diagnosed pulmonary arterial hypertension (PAH). ERA, endothelin receptor antagonist; PDE-5, phosphodiesterase-5; WHO FC, World Health Organization functional class

The treatment strategy for PAH in the study was performed a new treatment algorithm (Fig. 1). The treatment goal was peak VO_2_ > 15.0 mL/min/kg, which is stated as providing a “better prognosis” in the 2015 ESC/ERS guidelines for the diagnosis and treatment of pulmonary hypertension [3], and peak SBP during exercise > 120 mmHg. Patients were considered clinically stable when both treatment goals were reached. First-line treatment was an ERA, either bosentan or ambrisentan. Second-line treatment was the addition of PDE-5I, either sildenafil or tadalafil, with intravenous epoprostenol if needed. All other drugs, including beraprost, diuretics, digitalis, and anticoagulants, were permitted for use at the physician-in-charge’s discretion. Ultimately, physicians expert in treating PAH determined the optimal pharmacological therapy for each patient. All patients underwent cardiac catheterization at baseline and at 12 months, as well as CPX, laboratory measurement including brain natriuretic peptide (BNP), echocardiograms including tricuspid regurgitation pressure gradient (TRPG), and the completion of an HRQOL questionnaire at baseline and at 3, 6, and 12 months.

### Dosing regimens

Bosentan was given at an initial dose of 62.5 mg twice daily for 4 weeks, and was titrated to 125 mg twice daily thereafter. Ambrisentan was started at 5 mg once daily for 4 weeks and gradually increased to 10 mg once daily thereafter in the absence of side effects and with adequate tolerability. Sildenafil was titrated to a maximum of 20 mg three times daily. Tadalafil was started and maintained at 40 mg once daily. Given the individual variability in responses, the choice of drug and dose to be used in each patient was left to the discretion of the treating physician. All drug regimens were adjusted as necessary to limit side effects. Patients were informed of all the available treatment options. Whenever combination treatment was proposed, patients were informed of potential risks and side effects.

### Questionnaire

The SF-8 survey was used for the QOL assessment. The SF-8 has one question for each health domain and was developed as an alternative to the SF-36 survey (version 2) [21, 24], which is the most widely used patient-based health status survey. At baseline and at 3, 6, and 12 months, HRQOL was evaluated using the Japanese version of the SF-8 survey (standard version, recall at 4 weeks). Each of the SF-8 survey’s eight items represents one health profile dimension: general health perception, physical functioning, role functioning – physical, bodily pain, vitality, social functioning, mental health, and role functioning – emotional. The physical health component summary score (PCS) and mental health component summary score (MCS) were measured using the Norm-Based Scoring method, which is based on a large-scale population study conducted in Japan [17]. Higher scores on these subscales indicate better HRQOL.

### Exercise capacity

The 6MWD test is a measurement of the distance a person can walk in 6 min; in the present study, the 6MWD test was performed according to American Thoracic Society criteria [25]. Each patient underwent CPX at a progressively increasing work rate to maximal tolerance on a cycle ergometer. Oxygen and CO_2_ sensors were calibrated before each test by using gases with known O_2_, N_2_, and CO_2_ concentrations. The flow sensor was also calibrated before each test. All patients started at 10 W for a 3-min warm-up, followed by a 10-W/min ramp increment protocol. A 12-lead electrocardiogram was monitored continuously, and arm blood pressure was automatically measured every minute during exercise and throughout the recovery period. After achieving peak workload, all patients pedaled at a load of 0 W for a cool-down period of at least 2 min to prevent excess venous pooling. Test termination criteria comprised patient request, volitional fatigue, developed chest pain, ventricular tachycardia, suggestive ischemia (such as ≥2 mm of horizontal or down-sloping ST segment depression), second- or third-degree heart block, extreme hypertension, severe desaturation, and a drop in systolic blood pressure of ≥20 mmHg during exercise. A qualified exercise physiologist conducted each test under the supervision of a certified cardiologist. Respiratory gas-exchange variables, including VO_2_, CO_2_ output (VCO_2_), and minute ventilation (VE), were acquired continuously throughout CPX by using an Oxycon Pro ergospirometer (CareFusion Germany, Hӧchberg, Germany), and the gas-exchange data were obtained breath-by-breath. Peak VO_2_ and peak respiratory exchange ratio were defined as the highest 30-s average values obtained during the final stage of CPX. The VE/VCO_2_ slope was determined by means of a linear-regression analysis of the VE and VCO_2_ values obtained up to the respiratory compensation point during exercise.

### Statistical analysis

All data are expressed as mean ± 1 standard deviation. To evaluate the effect of sequential combination therapy, we used repeated-measures analysis of variance to assess the change of parameters over time in all patients, excluding two who died during the study. The association between HRQOL scores and pulmonary vascular resistance (PVR) or plasma BNP concentration at baseline were analyzed by using Spearman’s correlation coefficient. All statistical analyses were performed in SPSS 17.0 v. software (SPSS, Chicago, IL, USA). A *P* value of < 0.05 was considered statistically significant.

## Results

Patient characteristics at baseline are shown in Table 1. The mean age of the 30 patients (8 male, 22 female) was 57 ± 16 years. Mean pulmonary arterial pressure at baseline was 46 ± 12 mmHg. The percentages of patients that received the different sequential combination therapies are shown in Table 2. Two patients died during the study, one due to right heart failure at 4 months, the other to cardiac sudden death at 7 months.

**Table 1:** Patient baseline characteristics

Age (years)	57	±	16
Male, *n* (%)	8 (27)		
BMI (kg/m^2^)	23.3	±	4.8
WHO functional classification II/III/IV	5/15/10		
Idiopathic/porto/CTD	13/6/11		
Laboratory			
BNP (pg/mL) *	391 (69–558)		
Swan–Ganz data			
RAP (mm Hg)	6.1	±	4.2
Systolic PAP (mm Hg)	73.9	±	20.2
Diastolic PAP (mm Hg)	28.9	±	8.5
Mean PAP (mm Hg)	46	±	12
PAWP (mm Hg)	9	±	4.3
CO (L/min)	4.06	±	1.42
CI (L/min/m^2^)	2.68	±	0.93
PVR (Wood units)	11.3	±	6.8
SvO_2_ (%)	64.1	±	8.9
Pulmonary Function Testing			
FEV_1%_	76.9	±	11.2
%VC	87.4	±	19.8
%D_LCO_	61.2	±	26.4
Cardiopulmonary Exercise Testing			
Peak VO_2_ (mL/min/kg)	12.1	±	4.4
VE/VCO_2_ slope	55.1	±	20.3

Data are presented as mean ± SD or median (interquartile range)

*BSA* Body surface area, *BNP* Brain natriuretic peptide, *CI* Cardiac index, *CO* Cardiac output, *CTD* Connective tissue disease, *D*_*LCO*_ Carbon monoxide lung diffusing capacity, *FEV*_*1%*_ Forced expiratory volume, *PAP* Pulmonary arterial pressure, *PAWP* Pulmonary artery wedge pressure, *Porto* Portopulmonary hypertension, *PVR* Pulmonary vascular resistance, *RAP* Right atrial pressure, *SvO*_*2*_ mixed venous oxygen saturation, *UA* Uric acid, *VC* Vital capacity, *WHO* World Health Organization

* Normal range for BNP, < 18.4 pg/mL

**Table 2:** Pulmonary arterial hypertension-specific treatments used during the study

Medication	Baseline	3 months	6 months	12 months
	(*n* = 30)	(*n* = 30)	(*n* = 29)	(*n* = 28)
ERA	0	30 (100%)	29 (100%)	28 (100%)
PDE-5I	0	2 (7%)	23 (79%)	23 (82%)
Epoprostenol i.v.	0	0	0	1 (4%)

*ERA* Endothelin receptor antagonist, *PDE-5I* Phosphodiesterase-5 inhibitor, *i.v*. Intravenous administration

**Fig. 2:**
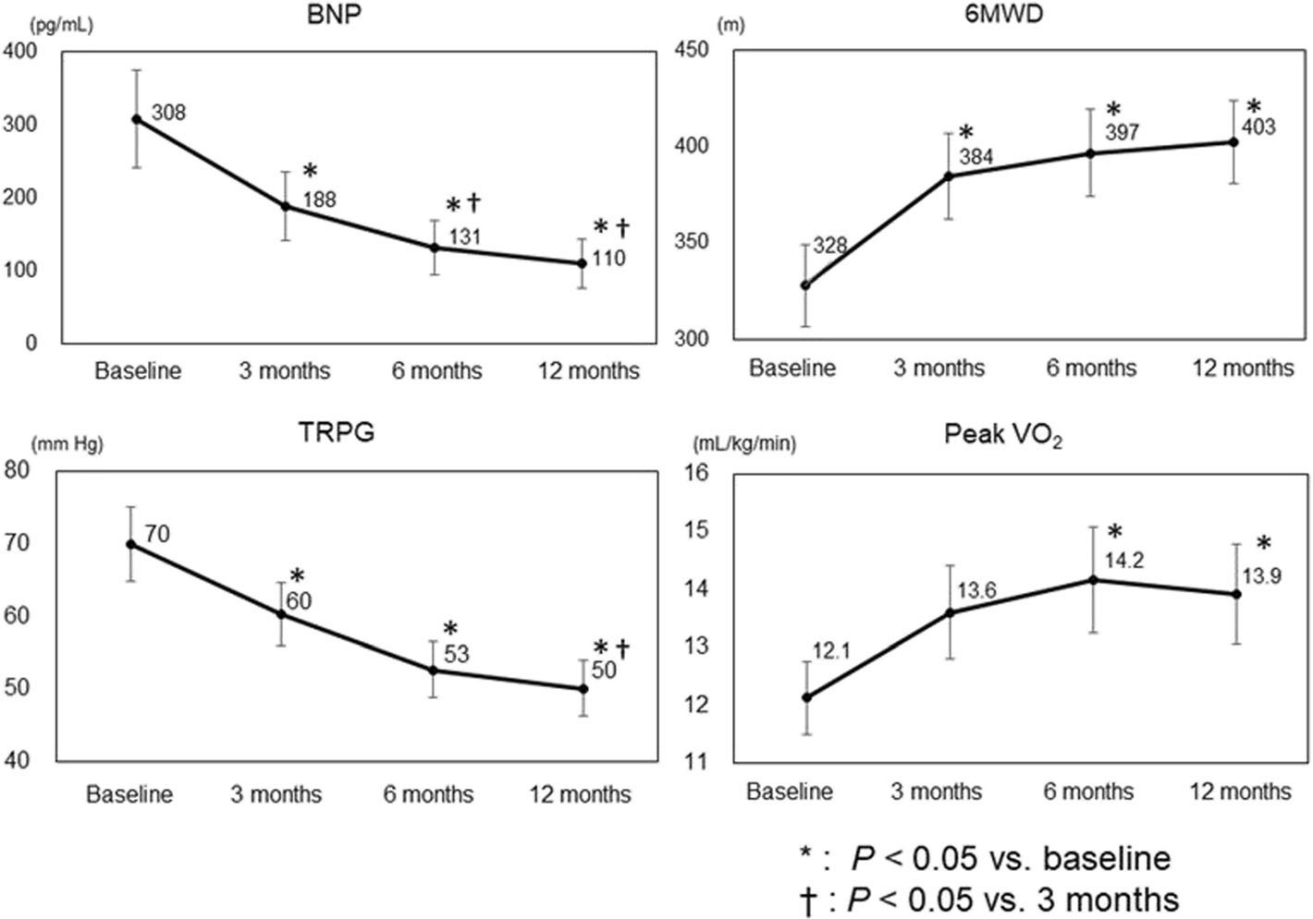
Parameter changes during the study period. BNP, brain natriuretic peptide; 6MWD, 6-min walking distance; TRPG, tricuspid regurgitation pressure gradient

At baseline, no patients had received an ERA or PDE-5I; at 3 months, all patients were receiving an ERA; and at 6 months, 79% of patients were receiving both an ERA and a PDE-5I. At 12 months, all patients were still receiving an ERA, 82% were receiving both an ERA and a PDE-5I, and 1 patient (4%) was receiving intravenous epoprostenol. The changes in plasma BNP, TRPG, and 6MWD at baseline and at 3, 6, and 12 months are shown in Fig. 2. Compared with baseline, BNP and TRPG were significantly reduced at 3 months, and were further reduced at 6 and 12 months. In contrast, 6MWD was significantly increased at 3 months, but was comparable at 3, 6, and 12 months. Compared with baseline, peak VO_2_ was significantly higher at 6 months. Mean pulmonary arterial pressure was significantly decreased from 45.6 mmHg at baseline to 37.7 mmHg at 12 months (*P* = 0.007). Mean PVR was significantly decreased from 10.1 Wood units at baseline to 5.6 Wood units at 12 months (*P* < 0.001).

**Fig. 3:**
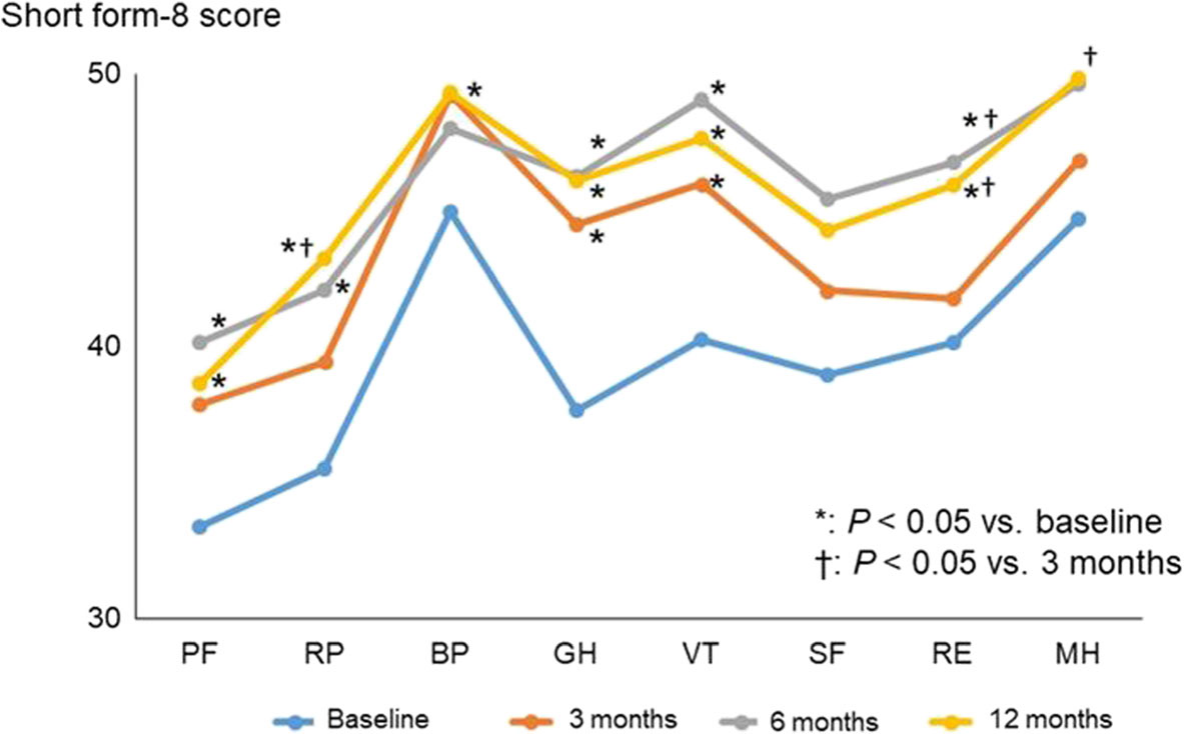
Changes in health-related quality of life score during the study period, as assessed by using the 8-item Medical Outcomes Study Short Form Health Survey. PF, physical functioning; RP, role functioning – physical; BP, bodily pain; GH, general health perception; VT, vitality; SF, social functioning; RE, role functioning – emotional; MH, mental health

**Fig. 4:**
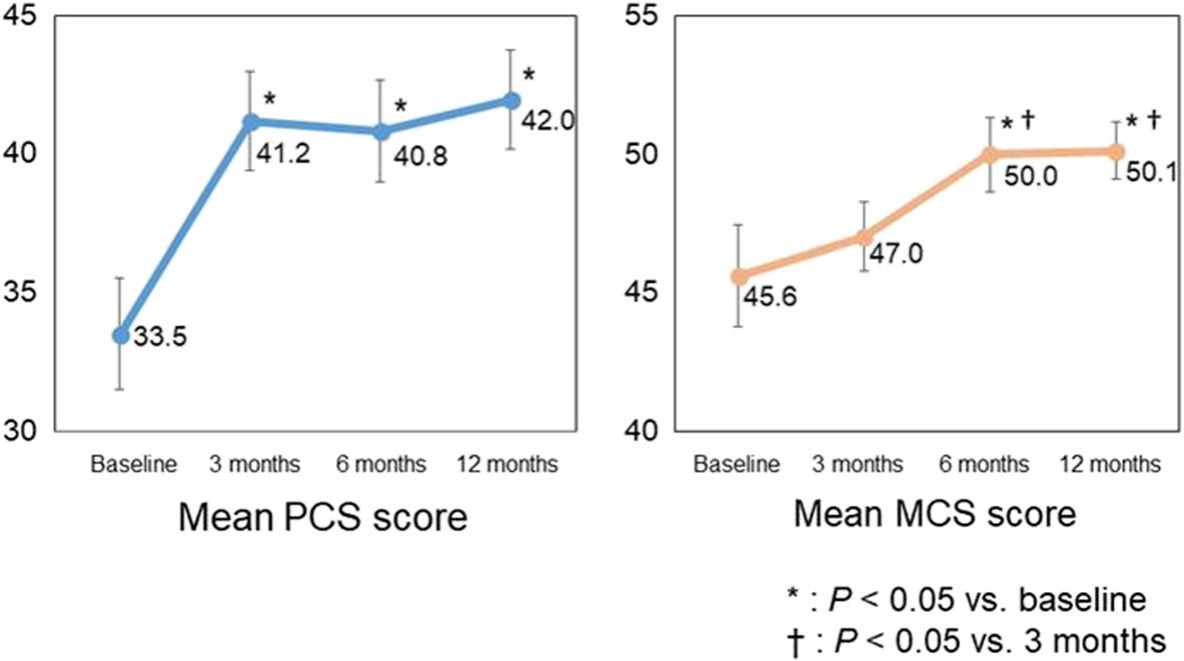
Changes in mean physical component summary (PCS) score and mental component summary (MCS) score during the study period

Changes in HRQOL score during the study period, as assessed by using the SF-8, are shown in Fig. 3. Treatment with sequential combination therapy significantly improved seven of the eight SF-8 domains except social functioning. Mean PCS scores were 33.5 at baseline, 41.2 at 3 months, 40.8 at 6 months, and 42.0 at 12 months, and mean MCS scores were 45.6, 47.0, 50.0, and 50.1, respectively (Fig. 4). The mean PCS score was significantly improved at 3 months compared with baseline (*P* = 0.003), but there were no significant differences in PCS score among 3, 6, and 12 months. In contrast, MCS was comparable at baseline and at 3 months (*P* = 0.47), but it was significantly improved at 6 and 12 months compared with baseline (*P* = 0.033 and *P* = 0.028, respectively). Furthermore, at baseline, PCS score was significantly correlated with PVR, plasma BNP, and peak VO_2_ (*r* = − 0.427, *r* = − 0.417, and *r* = 0.411, respectively), and MCS score was significantly correlated with peak VO_2_ (*r* = 0.299; Table 3). Significant negative correlations with PVR, plasma BNP and peak VO_2_ were found for five of the health profile dimensions (physical functioning, role functioning – physical, general health perception, vitality, and role functioning – emotional). Social functioning was significantly negatively correlated with plasma BNP and positively with peak VO_2_.

**Table 3:** Correlation between SF-8 health profile dimensions and PVR, BNP, and Peak VO_2_ at baseline

	PVR		Plasma BNP		Peak VO_2_	
	*r*	*P*	*r*	*P*	*r*	*P*
PF	−0.500	<0.001	−0.496	<0.001	0.501	<0.001
RP	−0.529	<0.001	−0.513	<0.001	0.657	<0.001
BP	0.050	0.754	−0.125	0.436	0.179	0.263
GH	−0.455	0.003	−0.327	0.037	0.396	0.010
VT	−0.390	0.012	−0.529	<0.001	0.531	<0.001
SF	−0.272	0.086	−0.349	0.025	0.427	0.005
RE	−0.337	0.031	−0.428	0.005	0.494	0.001
MH	−0.134	0.402	−0.213	0.181	0.211	0.186
PCS	−0.427	0.005	−0.417	0.007	0.411	0.003
MCS	−0.158	0.323	−0.280	0.076	0.299	0.037

*PVR* Pulmonary vascular resistance, *BNP* Brain natriuretic peptide

Health-related quality of life was assessed by using the Medical Outcomes Study Short Form Health Survey (SF-8), which consists of eight items, each representing one health profile dimension: *PF* Physical functioning; *RP* Role functioning – physical, *BP* Bodily pain, *GH* General health perception, *VT* Vitality, *SF* Social functioning, *RE* Role functioning – emotional; and *MH* Mental health. Physical component summary (PCS) and mental component summary (MCS) scores were compared with demographically adjusted United States norms and with historical controls

## Discussion

This is the first study to show the therapeutic effect on HRQOL over time under sequential combination therapy in patients newly diagnosed with PAH. Our goal-oriented treatment used CPX to guide therapeutic decisions, because CPX is noninvasive and has prognostic importance [7, 26]. To date, most investigations of PAH have examined hemodynamic parameters or cardiac function by means of cardiac catheterization at rest. However, these approaches are unsuitable for repeated assessment, and so they cannot be used to examine the efficacy of therapeutic regimens for the treatment of PAH. In addition, most patients with PAH become breathless only during exercise, and resting hemodynamic data are not enough to reflect dyspnea on effort in daily life or to predict prognosis. Indeed, QOL is associated with a prognostic marker in patients with PAH [23]. Therefore, we examined the efficacy of goal-oriented sequential combination therapy based on exercise capacity for the treatment of PAH by using the SF-8 health survey to assess HRQOL. We found that in patients newly diagnosed with PAH, HRQOL was gradually improved, which suggests that HRQOL assessment could provide useful information on the efficacy of therapeutic regimens for the treatment of PAH.

Previous studies have demonstrated that the HRQOL of PAH patients is severely reduced compared with that of healthy individuals [27]. PAH patients present with reduced physical mobility, marked dyspnea, and increased difficulties in social interactions [12, 13]. Indeed, our results show that HRQOL was low at baseline; however, it was gradually improved by goal-oriented sequential combination therapy based on exercise capacity. In addition, several of the health profile dimensions examined in the SF-8 health survey were significantly negatively correlated with both BNP and PVR, which are known to reflect PAH disease severity [28, 29]. Together, these results suggest that repeated assessment of HRQOL can be used to evaluate not only the severity of symptoms in daily life, but also therapeutic response, which is consistent with the findings of previous studies [30, 31].

The SF-8 health survey is a general, non-disease-specific QOL survey used to assess symptom severity [17]. The Japanese version of the SF-8 meets the standard criteria for content and for construct and criterion validity, based on a national survey of 1000 Japanese citizens conducted in 2002 [17]. SF-8 is a short version of SF-36 that permits comparison of HRQOL between patients with different diseases or comparison of health status between patients and the general population [17, 22, 32]. Compared with the SF-36, the SF-8, in combination with other questionnaires, is a more practical means of evaluating the actual condition of patients and their clinical responses because of its simplicity. In the present study, three health profile dimensions—role functioning – physical, role functioning – emotional, and mental health—were low at baseline, but were significantly improved at 6 months compared with 3 months. In addition, PCS improved before MCS. Depression is common in patients with PAH, at 55% [33]. Similarly, 6MWD was also significantly improved. Together, these results suggest that exercise tolerance and QOL can be improved with goal-oriented sequential combination therapy based on exercise capacity, possibly via improving hemodynamic state by noninvasive assessment of BNP and TRPG. Thus, physical performance was gradually improved, followed by mental status. This suggests that improvements in physical status positively influence mental status during sequential combination therapy in the medium-term. We speculate that this is because when patients notice the improvements in physical function brought about by the PAH-specific therapy, their confidence improves, which in turn brings about an improvement in their mental status.

In a previous study, patients with better baseline HRQOL had better long-term outcomes, as evidenced by a reduced risk of morbidity and mortality compared with patients whose PCS and MCS scores were less than the median baseline values [30]. In addition, PCS and MCS scores are both significantly associated with survival [31]. These data suggest that a primarily noninvasive treatment strategy and the use of combination therapy may yield acceptable results with regard to HRQOL in the majority of patients with PAH. The treatment strategy used included a period to check the side effects of each medication, and therapeutic decisions were based on physicians’ experience as well as on practicability and economic considerations. However, in clinical practice, we may need to comprehensively assess WHO functional class, 6MWD, and BNP response to therapy to decide whether additional therapy is required [34]. Therefore, further studies are needed to define the variables most useful for clinical decision-making as well as the treatment options that provide the best long-term results for patients with PAH.

The five patients who received only ERA improved to WHO-FC I or II and remained clinically stable without cardiac event; PDE-5Is were contraindicated in one of these patients because she was receiving amiodarone for the treatment of non-sustained ventricular tachycardia. The average peak VO_2_ among the ERA-only patients increased from 12.5 mL/kg/min at baseline to 17.4 mL/kg/min at 12 months, and the mean pulmonary arterial pressure decreased from 42.5 mmHg at baseline to 27.7 mmHg at 12 months. We believe that in this subgroup of PAH patients, monotherapy was the appropriate choice for at least 12 months, so our CPX-guided goal-based treatment strategy might avoid excess medication and cost while still providing adequate intervention by enabling treatment to be tailored to the individual patient.

At the start of the study, beraprost had already been used in 37% of the subjects. This drug is the first chemically stable, orally active prostacyclin analog approved for the treatment of PAH [35, 36], but its clinical effects subside after only half a year of use [37, 38]. Beraprost is currently approved for the treatment of PAH in Japan and South Korea, but worldwide the evidence level for the effectiveness of beraprost is low. Because hemodynamic parameters at baseline did not differ significantly between PAH patients who received beraprost and those who did not (data not shown), we excluded it from the goal-oriented sequential combination therapy.

There are several limitations to our study. First, the study was performed in a small number of patients and had short follow-up periods. However, since the study population included only patients newly diagnosed with PAH, we believe that the results obtained from this study are important for the treatment of newly diagnosed PAH. Second, we used the SF-8 survey instead of the SF-36; the SF-8 may not be appropriate for individual patient assessment and treatment decisions and may instead be better suited to population assessment [17, 21]. Also, we did not use PAH-specific HRQOL measures such as the ERS or REVEAL risk assessment calculators [34], which might produce different results compared with the SF-8. However, the SF-8 did detect a therapeutic effect of sequential combination therapy, which suggests that it can be used to monitor HRQOL in patients with PAH in the medium-term. Third, none of the patients underwent cardiac rehabilitation. Exercise training can have a positive impact on short-term functioning in certain patients with pulmonary hypertension [39]; therefore, if these patients had undergone cardiac rehabilitation, their exercise capacity might have been further improved.

## Conclusions

In patients with PAH, the PCS score improved relatively soon after the initiation of PAH-specific therapy, followed later by the MCS score. Goal-oriented sequential combination therapy based on exercise capacity might be useful for improving HRQOL in patients with PAH. HRQOL assessment provided useful information on the efficacy of this therapeutic regimen in patients with PAH.

## Data Availability

The datasets generated during and/or analyzed during the current study are available from the corresponding author on reasonable request.

## Abbreviations

6MWD: 6-min walking distance
BNP: Brain natriuretic peptide
CPX: Cardiopulmonary exercise testing
ERAs: Endothelin receptor antagonists
HRQOL: Health-related quality of life
MCS: Mental health component summary score
PAH: Pulmonary arterial hypertension
PCS: Physical health component summary score
PDE-5Is: Phosphodiesterase-5 inhibitors
PVR: Pulmonary vascular resistance
SF-8, − 36: 8- and 36-item Medical Outcomes Study Short Form Health Surveys
TRPG: Tricuspid regurgitation pressure gradient
VE: Minute ventilation
WHO: World Health Organization
